# Stress ulcer prophylaxis with a proton pump inhibitor versus placebo in critically ill patients (SUP-ICU trial): study protocol for a randomised controlled trial

**DOI:** 10.1186/s13063-016-1331-3

**Published:** 2016-04-19

**Authors:** Mette Krag, Anders Perner, Jørn Wetterslev, Matt P. Wise, Mark Borthwick, Stepani Bendel, Paolo Pelosi, Frederik Keus, Anne Berit Guttormsen, Joerg C. Schefold, Morten Hylander Møller, the SUP-ICU investigators

**Affiliations:** Department of Intensive Care 4131, Copenhagen University Hospital, Rigshospitalet, Blegdamsvej 9, 2100 Copenhagen, Denmark; Department of Intensive Care 4131 and Centre for Research in Intensive Care (CRIC), Copenhagen University Hospital, Rigshospitalet, Copenhagen, Denmark; Copenhagen Trial Unit, Centre for Clinical Intervention Research, Copenhagen University Hospital, Rigshospitalet, Copenhagen, Denmark; Department of Adult Critical Care, University Hospital of Wales, Cardiff, UK; Pharmacy Department, Oxford University Hospitals NHS Trust, Oxford, UK; Department of Intensive Care Medicine, Kuopio University Hospital, Kuopio, Finland; Department of Surgical Sciences and Integrated Diagnostics, IRCCS AOU San Martino IST, University of Genoa, Genoa, Italy; University of Groningen, Department of Critical Care, University Medical Center Groningen, Groningen, The Netherlands; Department of Anaesthesia and Intensive Care, Haukeland University Hospital and Clinical Institute 1 UiB, Bergen, Norway; Department of Intensive Care Medicine, University Hospital Inselspital, Bern, Switzerland

**Keywords:** Stress ulcer prophylaxis, Gastrointestinal bleeding, Intensive care unit, Critically ill, Randomised clinical trial, Placebo, Adverse event

## Abstract

**Background:**

Critically ill patients in the intensive care unit (ICU) are at risk of clinically important gastrointestinal bleeding, and acid suppressants are frequently used prophylactically. However, stress ulcer prophylaxis may increase the risk of serious adverse events and, additionally, the quantity and quality of evidence supporting the use of stress ulcer prophylaxis is low. The aim of the SUP-ICU trial is to assess the benefits and harms of stress ulcer prophylaxis with a proton pump inhibitor in adult patients in the ICU. We hypothesise that stress ulcer prophylaxis reduces the rate of gastrointestinal bleeding, but increases rates of nosocomial infections and myocardial ischaemia. The overall effect on mortality is unpredictable.

**Methods/design:**

The SUP-ICU trial is an investigator-initiated, pragmatic, international, multicentre, randomised, blinded, parallel-group trial of stress ulcer prophylaxis with a proton pump inhibitor versus placebo (saline) in 3350 acutely ill ICU patients at risk of gastrointestinal bleeding. The primary outcome measure is 90-day mortality. Secondary outcomes include the proportion of patients with clinically important gastrointestinal bleeding, pneumonia, *Clostridium difficile* infection or myocardial ischaemia, days alive without life support in the 90-day period, serious adverse reactions, 1-year mortality, and health economic analyses.

The sample size will enable us to detect a 20 % relative risk difference (5 % absolute risk difference) in 90-day mortality assuming a 25 % event rate with a risk of type I error of 5 % and power of 90 %. The trial will be externally monitored according to Good Clinical Practice standards. Interim analyses will be performed after 1650 and 2500 patients.

**Conclusion:**

The SUP-ICU trial will provide high-quality data on the benefits and harms of stress ulcer prophylaxis with a proton pump inhibitor in critically ill adult patients admitted in the ICU.

**Trial registration:**

ClinicalTrials.gov Identifier: NCT02467621.

## Background

Critically ill patients are at risk of stress-related gastrointestinal (GI) mucosal damage, ulceration and bleeding [1]. Endoscopic studies have shown that gastric erosions are present in up to 90 % of patients by the third day in the intensive care unit (ICU) [2, 3]. These lesions are, in the vast majority of patients, superficial and asymptomatic, but some can progress and result in overt and clinically important GI bleeding [4]. Clinically important GI bleeding in the ICU is a serious condition, with an estimated one- to four-fold increased risk of death and excess length of ICU stay of 4–8 days [1, 5]. It has been suggested that prophylaxis with acid suppressants reduces the risk of GI bleeding and hence the risk of death [6]. In this context, stress ulcer prophylaxis (SUP) was introduced and is recommended in international guidelines [7–10] and regarded as standard of care in the ICU [5, 11]. However, clinical research has not been able to confirm that SUP improves outcome [12]. A recent meta-analysis comprising 20 randomised clinical trials (RCTs) comparing proton pump inhibitors (PPIs) and/or histamine-2-receptor antagonists (H2RAs) versus placebo or no prophylaxis did not find any differences in patient important outcome measures between the SUP and the placebo/no prophylaxis groups [12]. Furthermore, concern has been expressed about potentially increased risks of side effects in patients receiving prophylactic treatment with acid suppressants [13–16]. The higher gastric pH in these patients may compromise host immunity and increase the risk of pneumonia and *Clostridium difficile* infection (CDI) [15, 17]. However, no meta-analyses of randomised trials have shown a significantly increased risk of nosocomial pneumonia when using SUP compared to placebo/no prophylaxis [12, 18]. Additionally, no trials have assessed the incidence of CDI in an ICU setting, but a recently published large cohort study found a 2–4 fold increased risk of CDI in adult mechanically ventilated patients receiving PPIs compared to H2RAs [19]. Studies conducted outside the ICU have demonstrated similar findings [20, 21]. Also, an association between the use of PPIs and an increased risk of cardiovascular events has been suggested [18, 22, 23].

Taken together, the balance between benefits and harms of SUP is unclear in critically ill patients in the ICU. The aim of the SUP-ICU trial is to assess the benefits versus harms of PPI (pantoprazole) in acutely ill adults in the ICU. We hypothesise that a PPI reduces the rates of GI bleeding, but increases the rates of nosocomial infections and myocardial ischaemia. The effect on overall mortality is, therefore, unpredictable.

## Methods

### Trial design

The SUP-ICU trial is an investigator-initiated, pragmatic, international, multicentre, randomised, blinded, parallel-group trial of SUP with a PPI versus placebo.

### Approvals

The trial is approved by the Danish Health and Medicine Agency (2015030166), the Committees on Health Research Ethics in the Capital Region of Denmark (H-15003141) and the Danish Data Protection Agency (RH-2015-3203695) and registered at ClinicalTrials.gov (Identifier: NCT02467621).

### Setting

European ICUs admitting adult patients.

### Population

#### Inclusion criteria

All adult (18 years or older) patients who are acutely admitted to the ICU with one or more risk factors for GI bleeding [5]:

- Shock (continuous infusion with vasopressors or inotropes, systolic blood pressure below 90 mmHg, mean arterial blood pressure below 70 mmHg or plasma lactate level 4 mmol/l or above)
- Acute or chronic intermittent or continuous renal replacement therapy (RRT)
- Invasive mechanical ventilation which is expected to last more than 24 hours
- Coagulopathy (platelets below 50 × 10^9^/l, or international normalised ratio (INR) above 1.5, or prothrombin time (PT) above 20 s) documented within the last 24 hours
- Ongoing treatment with anticoagulant drugs (prophylactic doses excluded)
- History of coagulopathy (platelets below 50 × 10^9^/l or INR above 1.5 or PT above 20 s within the 6 months prior to hospital admission)
- History of chronic liver disease (portal hypertension, cirrhosis proven by biopsy, computed tomography (CT) scan or ultrasound or history of variceal bleeding or hepatic encephalopathy)

#### Exclusion criteria

- Contraindications to PPIs (including intolerance of PPIs and treatment with atazanavir (anti-human immunodeficiency virus (HIV) medication))
- Current daily treatment with a PPI and/or a H2RA
- GI bleeding of any origin during current hospital admission
- Diagnosed with peptic ulcer during current hospital admission
- Organ transplant during current hospital admission
- Withdrawal from active therapy or brain death
- Fertile woman with positive test for urinary or plasma human chorionic gonadotropin (hCG)
- Consent according to national regulations not obtainable

### Trial medication

Enrolled patients will be randomised to receive either pantoprazole 40 mg (pantoprazole, Actavis, Gentofte, Denmark) or placebo, given once daily intravenously, from randomisation until ICU discharge or death for a maximum of 90 days. Identical vials with and without pantoprazole powder will be masked with a full covering label. The nurse caring for the patient will have access to an electronic medication distribution system, which allows the allocation of the appropriate vial to the patient. The nurse will add 10 ml of sodium chloride to the vial, shake it, and administer the contents intravenously to the patient. As the powder immediately dissolves to a colourless fluid it will not be possible to distinguish dissolved pantoprazole in sodium chloride from sodium chloride alone.

### Outcome measures

#### Primary outcome measure

All-cause mortality 90 days after randomisation

#### Secondary outcome measures

- Proportion of patients with one or more of the following adverse events during ICU stay: clinically important GI bleeding, pneumonia, CDI, or acute myocardial ischaemia
- Proportion of patients with clinically important GI bleeding during ICU stay
- Proportion of patients with one or more infectious adverse events (pneumonia or CDI) during ICU stay
- Days alive without use of mechanical ventilation, RRT or circulatory support in the 90-day trial period
- Number of serious adverse reactions (SARs) during ICU stay
- Mortality 1 year after randomisation
- A health economic analysis will be performed. The analytic details will be based on the results of the trial and specified at that time (cost-benefit versus cost-minimisation analyses)

The specific elements of the composite outcomes will be reported in the primary publication.

### Definitions

See Appendix 1.

### Screening

All patients referred to a participating clinical trial site will be considered for participation (screened). Patients will be eligible if they fulfil all of the inclusion criteria and none of the exclusion criteria listed. Inclusion and exclusion of patients (including reasons for exclusion) will be reported according to the Consolidated Standards of Reporting Trials (CONSORT) statement [24].

### Randomisation

Staff at trial sites will have 24-hour access to web-based central randomisation allowing immediate and concealed allocation of trial medication. Randomisation will be performed in blocks with varying block sizes according to the generation of the allocation sequence by the Copenhagen Trial Unit (CTU) [25]. A unique patient identification number will be entered into the system to ensure that the patient is not randomised twice. In addition, each patient will be allocated a unique patient number (screening number).

### Blinding

The allocated trial medication will be blinded to the patient, the clinical staff caring for the patient, the investigators, the outcome assessors, the data manager, the statistician conducting the analyses, and the writing committee when drafting the abstract for the primary publication.

An independent company (Nomeco Clinical Trial Supply Management (CTSM) [26]) will handle masking, coding and distribution of the vials containing the investigational medicinal product (IMP)/placebo. A computer programme will generate the coding list (CTU) with numbers for the vials. Each trial site will have a sufficient number of vials to be allocated to participating patients. This will ensure that the patient only receives the trial intervention they are randomised to receive.

### Safety

Patients can be withdrawn from the trial if:

- A clinical indication for treatment with a PPI/H2RA arises (GI bleeding and/or ulcer/gastritis/varices verified endoscopically). Patients will receive treatment for GI bleeding according to local standards
- Another clinical indication for withdrawal than the above mentioned (judged by responsible clinician or local investigator)
- A SAR/suspected unexpected serious adverse reaction (SUSAR) occurs (see below)
- The patient or next of kin withdraws consent

The independent Data Monitoring and Safety Committee (DMSC) can recommend pausing or stopping the trial. Details are provided in Appendix 2.

#### Serious adverse reactions

Adverse reactions are specified in the product characteristics of pantoprazole. The following conditions related to the intervention will be considered SARs:

- Anaphylactic reactions
- Agranulocytosis
- Pancytopenia
- Acute hepatic failure
- Stevens-Johnson syndrome and toxic epidermal necrolysis
- Interstitial nephritis
- Angioedema (Quincke’s oedema)

The occurrence of SARs will be recorded daily in the electronic case report form (eCRF) during ICU stay and the distribution of SARs in the two groups will be compared by the DMSC at the interim analyses. During the trial the sponsor will send a yearly report to the ethics committees and medicine agencies.

SUSARs are defined as serious adverse events (SAEs) not described in the product characteristics for pantoprazole. SUSARs will be reported by the trial site investigators to the sponsor within 24 hours. The sponsor will report any SUSAR to the medicine agency within 7 days.

SAEs will not be recorded as an entity because the majority of ICU patients will experience a number of SAEs during their critical illness. SAEs will be captured in the secondary outcome measures.

### Patient withdrawal

Patients who are withdrawn from the trial intervention will be followed-up and included in the intention-to-treat analysis. Patients may be withdrawn from the trial according to national consent regulations. In order to limit the amount of missing data, as much data as possible from each patient will be collected. All randomised patients will be reported, and all data available with consent will be used [27].

Patients who are transferred to another ICU will be regarded as discharged from the ICU unless the new ICU is an active SUP-ICU trial site. If so, the allocated trial intervention will be continued. All patients transferred to another ICU will be followed-up for the primary outcome measure.

### Statistics

A predefined analysis plan will be prepared and published before data analysis.

The primary analysis will include the intention-to-treat population comparing mortality 90 days after randomisation in the two groups by binary logistic regression analysis with adjustment for stratification variables: site and active haematological cancer. A secondary analysis will be performed adjusting for stratification variables together with other known major prognostic co-variates: age, baseline Sequential Organ Failure Assessment (SOFA) score, and type of admission (medical, elective surgery or emergency surgery). A sensitivity analysis will be conducted including the per-protocol population, excluding patients with a major protocol violation (patients who did not receive the allocated trial intervention at all, patients who did not receive the trial intervention for at least 2 days in a row, treatment with a PPI or a H2RA without clinical indication and withdrawal from trial intervention). The prevalence and pattern of missing values will be collected and analysed according to the predefined statistical analysis plan. If missingness exceeds 5 % and data is not missing completely at random (Little’s test <0.05) multiple imputation with at least 10 imputations will be performed, and the primary result of the analysis will be from the aggregated intervention effects from the imputed datasets. All statistical tests will be two-tailed and *P* < 0.05 will be considered statistically significant.

#### Sample size estimation

Assuming a baseline 90-day mortality of 25 % [5] (see Appendix 3), *α* = 0.05 (two-sided), and *β* = 0.1, 3350 patients (2 × 1675) will be needed to show a 20 % relative risk reduction (RRR) or increase (RRI) corresponding to a 5 % absolute risk reduction or risk increase in the primary outcome measure.

#### Interim analyses

Interim analyses will be performed after 1650 and 2500 patients. The DMSC may recommend pausing or stopping the trial if the group difference in the primary outcome measure, SARs or SUSARs is found at the interim analyses with statistical significance levels adjusted according to the LanDeMets group sequential monitoring boundaries based on the O’Brien-Fleming alpha-spending function, or otherwise finds that the continued conduct of the trial clearly compromises patient safety.

### Data registration

Data will be entered into a web-based eCRF (CTU) by trial or clinical personnel. From the eCRF the trial database will be established. Paper case report forms (CRFs) will be used in case of technical difficulties with the eCRF. Details on data collection are shown in Appendix 1.

### Data handling and retention

Data will be handled according to the national data protection agencies. All original records (including consent forms, CRFs, SUSAR reports and relevant correspondences) will be retained at trial sites or the CTU for 15 years to allow inspection by the Good Clinical Practice (GCP) Unit or local authorities. The trial database will be maintained for 15 years and anonymised if requested by the authorities.

### Monitoring

The trial will be externally monitored according to a monitoring plan developed in collaboration with the GCP Unit in Copenhagen, which will coordinate the monitoring done by local GCP Units and/or monitors in all countries. Trial site investigators will give access to source data. A centralised day-to-day monitoring of the eCRF will be done by the coordinating investigator or her delegates.

### Ethical justification

The trial will adhere to the latest version of the Helsinki Declaration [28] and the national laws in the participating countries. Inclusion will start after approval by the ethical committees, medicines agencies and data protection agencies.

Stress ulceration is a condition often seen in critically ill patients in the ICU [1]. The majority of patients will be temporarily incompetent because of severe illness or as a consequence of the treatment, including sedation. We cannot perform the trial in competent patients because less sick (and thus competent) patients do not suffer from stress ulcers. Patients requiring acute treatment in the ICU, e.g. mechanical ventilation, are in an acute life-threatening condition and it would expose the patient to great risk not to initiate the necessary treatment in order to obtain informed consent. To conduct clinical trials with the goal of improving the outcome for ICU patients at risk of stress-related GI bleeding, it is necessary to randomise and enrol patients before obtaining their informed consent. Informed consent will be obtained from all participants or representatives according to the national regulations. The process leading to the achievement of consent may differ in the participating countries, but will be described and be in compliance with all applicable local regulations.

No biological material will be collected for the trial; thus, no bio-bank will be formed.

### Enrolment

Patients from Denmark, Finland, Italy, The Netherlands, Norway, Switzerland and the United Kingdom are expected to participate in the trial. The trial will be initiated in Denmark in January 2016 followed by the other countries when national approvals are obtained. The trial is expected to recruit patients during a 2-year period.

### Trial management and organisation

The trial is part of the SUP-ICU research programme [29] and is supported by the Centre for Research in Intensive Care (CRIC) and the CTU.

A Steering Committee has been formed consisting of all national principal investigators and a Management Committee (see Appendix 4). The Steering Committee will manage and coordinate the trial centrally.

A local research team consisting of a principal investigator and a trial coordinator will manage and coordinate the trial locally. The principal investigator has the responsibility for data collection and maintenance of trial documentation.

Co-enrolment of participants in other interventional trials has to be approved by the SUP-ICU Steering Committee, but is generally allowed.

### Publication

Upon trial completion the main manuscript with trial results, whether positive, negative or neutral, will be submitted for peer-review to one of the major clinical journals. Furthermore, the results will be published at the SUP-ICU web page [29].

The Steering Committee will grant authorship depending on personal input according to the Vancouver Principles. The DMSC and investigators not qualifying for authorship will be acknowledged with their names under the ‘SUP-ICU trial investigators’ in an appendix to the final manuscript.

### Data sharing

According to the recommendations from the Institute of Medicine and the Scandinavian Trial Alliance a clean file dataset used for final analysis of the main results of the trial, the statistical analysis plan, a variable explanation, and the protocol will be made publicly accessible in an anonymised form 2 years after the last follow-up of the last patients [30].

### Timeline

2014–2015: applications for funding, ethical committees and medicine agencies, development of an eCRF, development of monitoring plan and education of clinical staff

2016–2017: inclusion of patients

2018: data analyses, writing and submission of the main manuscript for publication

2021: data sharing according to the CRIC contract between partners [31]

### Collaborators

The trial has been developed and conducted in collaboration with the Scandinavian Critical Care Trial Group (SCCTG). The trial is administered by the CRIC [31]. The CTU has developed the eCRF in close collaboration with the Steering Committee. The web-based randomisation system and the system for allocation of trial medication have been developed and administered by the CTU. Pharma-Skan ApS produces the placebo vials and Nomeco CTSM masks and distributes trial medication to all sites.

### Finances

The trial is funded by the Innovation Fund Denmark and supported by the Aase and Ejnar Danielsens Foundation, the Ehrenreichs Foundation, the Scandinavian Society of Anaesthesia and Intensive Care Medicine (SSAI), the Danish Society of Anaesthesiology and Intensive Care Medicine (DASAIM), the Danish Medical Association, and the European Society of Intensive Care Medicine. Patient insurances will be sought financed from public and private funds. The funding sources will have no influence on trial design, trial conduct, data handling, data analysis or publication.

## Discussion

### Trial rationale

Clinical trials have suggested that there is a reduction in the incidence of GI bleeding among ICU patients receiving SUP compared with ICU patients receiving placebo or no prophylaxis [3, 32–38]. Based on this research conducted 15–20 years ago, and because of potentially increased mortality and morbidity in patients with clinically important bleeding, SUP is recommended as a standard of care in critically ill patients [7]. Around 75 % of critically ill patients in the ICU receive an acid suppressant during their ICU stay and PPIs are the most frequently used agents [5]. However, the quantity and quality of evidence supporting a reduction in clinically important GI bleeding and mortality with these agents is low [12]. Importantly, it has been suggested that PPIs may increase the risk of pneumonia, CDI, and acute myocardial ischaemia, and SUP may, in the worst case scenarios, increase mortality [13–16]. Taken together, SUP with a PPI is standard of care in ICUs worldwide but has never been tested in large high-quality clinically placebo-controlled trials. As a consequence, PPIs have been used as SUP for several years without convincing evidence of improved outcome.

### Population

The population in this trial constitutes adult patients acutely admitted to the ICU with one or more risk factors for GI bleeding [5].

### Intervention

In recent years a PPI has been considered the drug of choice in the management of most acid-related GI disorders [39]. The superior efficacy of PPIs over H2RAs has been demonstrated in various GI disorders, including peptic ulcer disease [39], and randomised trials and meta-analyses have assessed PPIs compared to H2RAs as SUP in the ICU. A recently published meta-analysis by Alhazzani et al. (14 trials, 1720 patients) compared a PPI and a H2RA [40], and found that a PPI was more efficient in reducing clinical important and overt GI bleeding, but no differences were shown regarding mortality, length of stay or incidence of pneumonia [40].

In most countries PPIs are more frequently used as SUP than H2RAs [5]. Since PPIs are considered equally effective, and pantoprazole is the most frequently used PPI [5], we chose this as the intervention.

### Comparator

As described in the previous section, it has been suggested that a PPI is superior to a H2RA in the prevention of clinically important and overt GI bleeding. However, before comparing different SUP agents we need firm evidence of SUP being superior to placebo. This information is currently not available [12].

### Outcome

Assessing mortality as the primary outcome has a number of advantages. First, mortality has not been the primary outcome of previous trials and we are sceptical that previous trials have collected high-quality data on mortality other than short-term mortality (ICU/hospital) [12]. Second, nearly all previous trials assessing PPIs or H2RAs as SUP have high risks of bias [12]. We know that trials with high risks of bias tend to overestimate benefit and underestimate harm [41]. Accordingly, previous trial results might be biased and even though they seem to find a neutral effect on mortality this may be a biased estimate actually concealing excess mortality in the SUP group. Third, meta-analysis of previous trials did not reach a realistic information size so even neutral mortality estimates may be misleading [12]. Fourth, as a consequence of the 6S trial [42], where we found that bleeding was associated with death and that death was partly mediated by bleeding (and renal insufficiency), it appears less likely that there should be a clinically significant reduction in GI bleeding (if PPIs do prevent GI bleeding) without any effect on mortality [43]. Consequently, assessing mortality as the primary outcome measure gives the opportunity to weigh up potential benefits and harms.

### Sample size

It is difficult to produce reliable sample size estimations according to anticipated effects on GI bleeding because we have no reliable control group data due to the widespread use of PPIs [5]. As a consequence, it has been necessary to calculate sample size estimations given that something may change if we stop/avoid using PPIs until GI bleeding actually happens (see Appendix 3). The chosen intervention effect of 20 % RRR or RRI of the primary outcome may seem high, but in a population with septic shock or in, e.g. patients after cardiac arrest, a 20 % hazard ratio reduction corresponds to 1 month of extra median survival in patients with a median survival time of approximately 5 months. Furthermore, 3350 patients included in a low-risk-of-bias trial would make a huge contribution to existing evidence, more than doubling the number of randomised patients and providing trial results with low risk of bias on mortality and SAEs. Additionally, trial sequential analysis (TSA) [44, 45] of existing trials (*n* = 16) has shown that 34 % (1584 patients) of the required information size to detect or reject a 20 % RRR has been accrued; corresponding to a required information size of 4575 patients [12] (see Appendix 5). Consequently, there is an information gap of around 3000 patients assuming a 20 % RRR in mortality. With the inclusion of an additional 3350 patients it is expected that the pooled effect will cross the boundary for benefit/harm or the boundary for futility.

However, no single trial, whether large or well-conducted, gives the final answer and the SUP-ICU trial will not be an exception. Thus, existing meta-analyses of SUP should be updated with the SUP-IUC trial results.

### Strengths

The SUP-ICU trial is a large multicentre clinical trial designed to provide high-quality data with low risk of bias. The trial is monitored according to GCP standards, and before data analyses a statistical analysis plan will be available. Furthermore, the strengths include concealed group assignment, blinding of the patient, the clinical staff caring for the patient, the investigators, the outcome assessors, the data manager, and the trial statistician. The trial design is pragmatic with routine practice maintained except from prescription of SUP; with resulting high generalisability.

Prior to designing the trial we have thoroughly described the available evidence in systematic reviews and a meta-analysis with TSA [12, 46]. Determining the incidence of GI bleeding in critically ill patients in the ICU is complicated by varying definitions of the outcome, difficulties in measuring the outcome, and differences in case mix. To make sure the available data on GI bleeding and risk factors were valid and up-to-date we conducted a large international observational study assessing the incidence of GI bleeding, risk factors for GI bleeding, and the use of SUP in more than 1000 adult critically ill patients in the ICU [5].

### Limitations

As already described in previous sections the sample size estimation is based on estimates, as we do not have valid data describing mortality among patients with risk factors for GI bleeding not treated with a PPI due to the widespread use of acid suppressants. The power for even major effects on each of the possible side effects (pneumonia, CDI and acute myocardial ischaemia) may be reduced, but it will still make a large contribution to our knowledge on these outcomes that may seriously question, overthrow or confirm what we know so far. Furthermore, assessing the potential side effects as a composite outcome measure will increase the power. Additionally, there is a risk of excluding high-risk patients as patients already receiving daily treatment with a PPI or a H2RA cannot be enrolled in the trial due to the risk of discontinuing a therapy for another indication, e.g. history of peptic ulcer. The definition of overt GI bleeding includes haematochezia which might occur from a lower GI bleeding source not affected by PPI, e.g. colonic bleeding. Finally, we do not assess the use of a H2RA or other SUP agents and will not be able to draw conclusions about these drugs.

### Perspective

The SUP-ICU trial will provide important high-quality data and the results will inform clinicians, guideline committee members and policy-makers on the use of SUP in ICU patients. Together with existing data the trial will establish a more solid evidence base for the use of a prophylactic PPI in critically ill patients in the ICU.

### Trial status

Recruiting. First patient planned for inclusion in January 2016.

## Figures and Tables

**Fig. 1:**
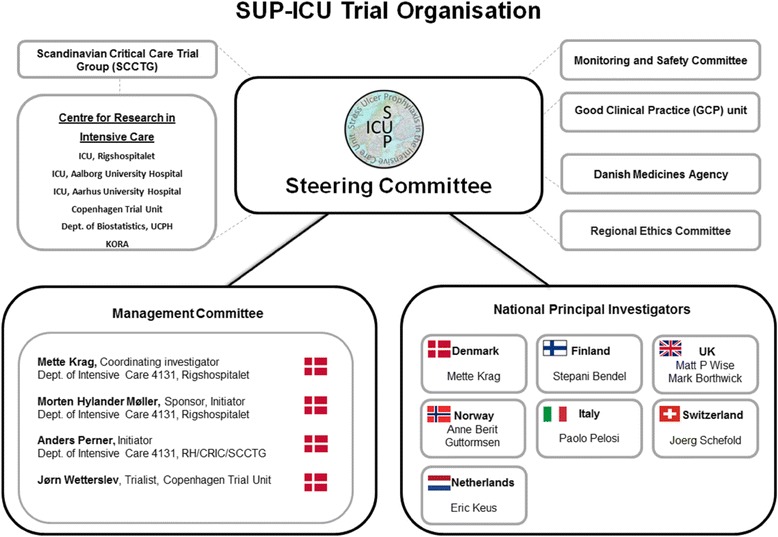
ᅟ

**Fig. 2:**
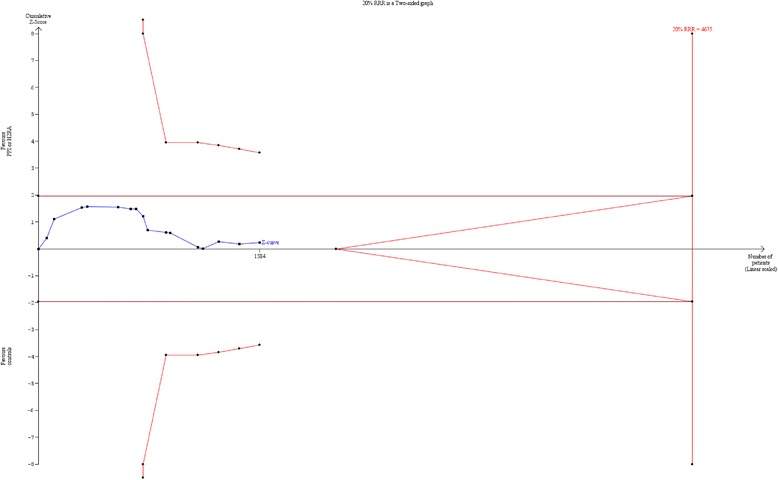
Trial sequential analysis of all-cause mortality (16 trials)

## Appendix 1. Definitions used in the SUP-ICU trial

### Definition of stratification variables

Site: all participating intensive care units (ICUs) will be assigned a number identifying the department.

Haematological malignancy includes any of the following:

Leukemia: acute lymphoblastic leukemia (ALL), acute myelogenous leukemia (AML), chronic myelogenous leukemia (CML), chronic lymphocytic leukemia (CLL).

Lymphoma: Hodgkin’s disease, non-Hodgkin lymphoma (e.g. small lymphocytic lymphoma (SLL), diffuse large B-cell lymphoma (DLBCL), follicular lymphoma (FL), mantle cell lymphoma (MCL), hairy cell leukemia (HCL), marginal zone lymphoma (MZL), Burkitt’s lymphoma (BL), post-transplant lymphoproliferative disorder (PTLD), T-cell prolymphocytic leukemia (T-PLL), B-cell prolymphocytic leukemia (B-PLL), Waldenström’s macroglobulinemia, other NK- or T-cell lymphomas.

Multiple myeloma/plasma cell myeloma.

### Definition of inclusion criteria

Acute admission to the ICU: a non-planned admission. It does not include planned recovery after surgery or similar planned admissions. ICU admission does not include admissions to semi-intensive care, intermediate intensive care or similar beds.

Age: the age of the patient in whole years at the time of randomisation. The age will be calculated from date of birth.

Shock: at least one of the following:

- Systolic pressure below 90 mmHg
- Mean arterial pressure below 70 mmHg
- Use of vasopressors or inotropes (norepinephrine, epinephrine, phenylephrine, vasopressin or dopamine, dobutamine, milirinone or levosimendan)
- Lactate level 4 mmol/l or above

Renal replacement therapy: acute or chronic intermittent or continuous renal replacement therapy.

Patients with expected duration of invasive mechanically ventilation longer than 24 hours: the treating clinician estimates that the patient will be invasively mechanically ventilated for more than 24 hours. When there is doubt about this forecast the patient should be enrolled.

Coagulopathy: platelets below 50 × 10^9^/l or international normalised ratio (INR) above 1.5 or prothrombin time (PT) above 20 s documented within the last 24 hours.

Treatment with anticoagulant drugs: ongoing treatment with: dipyridamole, vitamin K antagonists, ADP-receptor inhibitors, therapeutic doses of low-molecular-weight heparin, new oral anticoagulant drugs, intravenous direct thrombin (II) inhibitors and similar drugs.

Acetylsalicylic acid (all doses) and low-molecular-weight heparin in prophylactic doses are *not* included.

History of coagulopathy: coagulopathy defined as platelets below 50 × 10^9^/l *and/or* INR above 1.5 *and/or* PT above 20 s within the 6 months prior to hospital admission.

History of chronic liver disease: portal hypertension, cirrhosis proven by biopsy, CT scan or ultrasound, history of variceal bleeding or hepatic encephalopathy in the past medical history.

### Definition of exclusion criteria

Contraindications to proton pump inhibitors (PPIs): any history of intolerance to PPIs or additives or treatment with atazanavir (HIV medication).

Ongoing treatment with PPIs and/or histamine-2-receptor antagonists (H2RAs): ongoing, documented daily treatment with the drugs in the patient charts.

Gastrointestinal (GI) bleeding during current hospital admission: GI bleeding of any origin (both upper and lower) documented in the patient charts.

Peptic ulcer: peptic ulcer confirmed by endoscopy or other method during current hospital admission.

Organ transplant: any kind of organ transplant during current hospital admission.

Withdrawal from active therapy or brain death: patients where withdrawal or brain death is documented in the patient charts.

Known pregnancy: fertile woman with a positive test for urinary or plasma human chorionic gonadotropin (hCG).

Consent not obtainable according to national regulations: patients where the clinician or investigator is unable to obtain the necessary consent before inclusion of the patient according to the national regulations.

### Definition of baseline variables

Sex: the genotypic sex of the patient.

Age: defined in inclusion criteria.

Date of admission to hospital: the date of admission to the first hospital the patient was admitted to during the current hospital admission.

Elective surgery: surgery during the current hospital admission scheduled 24 hours or more in advance.

Emergency surgery: surgery during current hospital admission that was added to the operating room schedule 24 hours or less prior to that surgery.

Medical admission: when no surgery has been performed during the current hospital admission *or* surgery has been performed more than 1 week prior to ICU admission.

Treatment with anticoagulants at hospital admission and at ICU admission: anticoagulants are defined in the inclusion criteria.

Treatment with non-steroidal anti-inflammatory drugs (NSAIDs) and acetylsalicylic acid at hospital admission: treatment with all doses of these drugs at hospital admission.

Treatment with intravenous thrombolysis: treatment with all kinds of intravenous thrombolysis within 3 days prior to randomisation.

Coagulopathy: defined in the inclusion criteria.

Treatment of suspected or confirmed *Clostridium difficile* infection (CDI) during current hospital admission.

Coexisting illnesses must have been present in the past medical history prior to ICU admission and are defined as follows:

- Chronic lung disease: chronic obstructive pulmonary disease (COPD), asthma or other chronic lung disease or treatment with any relevant drug indicating this at admission to hospital
- Previous myocardial infarction: history of myocardial infarction
- Chronic heart failure: New York Heart Association (NYHA) functional class III–IV. NYHA class III: the patient has marked limitations in physical activity due to symptoms (fatigue, palpitation or dyspnoea) even during less than ordinary activity (walking short distances 20–100 m or walking up one flight of stairs). The patient is only comfortable at rest. NYHA class IV: the patient is not able to carry out any physical activity (without discomfort (fatigue, palpitation or dyspnoea). Symptoms are present even at rest and the patient is mostly bedbound
- History of chronic renal failure: need of any form of chronic renal replacement therapy within the last year
- Liver disease: defined in baseline variables
- History of coagulopathy: defined in baseline variables
- Immunosuppression: patients treated with at least 0.3 mg/kg/day of prednisolone equivalent for at least 1 month in the 6 months prior to ICU admission
- Metastatic cancer: proven metastasis by surgery, CT scan or any other method
- Haematological malignancy: defined as stratification variable
- AIDS: HIV-positive patients with one or more HIV-defining diseases such as *Pneumocystis jerovechii* pneumonia, Kaposi’s sarcoma, lymphoma, tuberculosis or toxoplasma infection

The Simplified Acute Physiology Score (SAPS II) is based on the most extreme (highest or lowest) values from 24 hours prior to randomisation. The score consists of 17 variables: 12 physiological variables, age, type of admission, and 3 variables related to underlying disease, to give a total score ranging from 0 to 163, with higher scores indicating greater illness severity. The score will be calculated from data from the 24 hours prior to randomisation.

The Sequential Organ Failure Assessment (SOFA) score will be calculated from raw physiology and treatment data from the 24 hours prior to randomisation. The SOFA score consists of weightings for six organ systems to give a total score ranging from 0 to 24, with higher scores indicating a greater degree of organ failure.

### Definition of daily collected variables

Delivery of trial medication: confirmation of administration of the trial drug.

Treatment with a PPI or a H2RA: prescription of any of these drugs in any dose (major protocol violation if the treatment is initiated (e.g. as prophylaxis) without clinical indication (e.g. GI bleeding).

Mechanical ventilation: invasive and non-invasive mechanical ventilation including continuous mask continuous positive airway pressure (CPAP) or CPAP via a tracheotomy. Intermittent CPAP is *not* mechanical ventilation.

Circulatory support: continuous infusion of vasopressor or inotrope (norepinephrine, epinephrine, phenylephrine, vasopressin or dopamine, dobutamine, milirinone or levosimendan).

Renal replacement therapy: any form of renal replacement therapy on this day. In patients receiving intermittent renal replacement therapy days between treatments are included.

Clinically important GI bleeding, onset of pneumonia, CDI, and acute myocardial ischaemia in the ICU are defined as outcomes.

Treatment with enteral feeding: any dose of enteral feeding (including oral nutritional intake) during the day.

Units of red blood cells: cumulated number of units of red blood cells transfused during the day.

Serious adverse reactions (SARs) are defined below.

### Definition of bleeding variables

Confirmed diagnosis: diagnosis/origin of bleeding confirmed by endoscopy or other method.

Verification of ulcer/gastritis/bleeding oesophageal varices: confirmation of one of the three specific diagnoses by endoscopy or other method.

Haemostasis achieved or attempted: documentation in patient charts of haemostasis achieved or attempted by endoscopy, open surgery or coiling.

### Definitions of outcome measures

Primary outcome:

90-day mortality: death from any cause within 90 days following the day of randomisation.

Secondary outcomes:

proportion of patients with one or more of the following adverse events: clinically important GI bleeding, pneumonia, CDI, and acute myocardial ischaemia. The events are defined as follows:

Clinically important GI bleeding: overt GI bleeding* and at least one of the following four features within 24 hours of GI bleeding (in the absence of other causes) in the ICU:

1. Spontaneous drop of systolic blood pressure, mean arterial pressure or diastolic blood pressure of 20 mmHg or more
2. Start of vasopressor or a 20 % increase in vasopressor dose
3. Decrease in haemoglobin of at least 2 g/dl (1.24 mmol/l)
4. Transfusion of two units of packed red blood cells or more

*Overt GI bleeding: haematemesis, coffee ground emesis, melaena, haematochezia or bloody nasogastric aspirate.

Pneumonia: episodes of newly confirmed pneumonia according to the modified CDC criteria [47]:

- Two or more serial chest radiographs with at least one of the following (one radiograph is sufficient for patients with no underlying pulmonary or cardiac disease):
  1. New or progressive and persistent infiltrate
  2. Consolidation
  3. Cavitation
- *and* at least one of the following:
  1. Fever (above 38 °C) with no other recognised cause
  2. Leucopoenia (white cell count below 4 × 10^9^/l) or leucocytosis (white cell count above 12 × 10^9^/l)
- *and* at least two of the following:
  1. New onset of purulent sputum or change in character of sputum, or increased respiratory secretions or increased suctioning requirements
  2. New onset or worsening cough, or dyspnoea, or tachypnoea
  3. Rales or bronchial breath sounds
  4. Worsening gas exchange (hypoxaemia, increased oxygen requirement, increased ventilator demand)

CDI: treatment with antibiotics (enteral vancomycin, intravenous or enteral metronidazole, enteral fidaxomicin) for suspected or proven CDI.

Acute myocardial ischemia: ST elevation myocardial infarction, non-ST elevation myocardial infarction or unstable angina pectoris according to the criteria in the clinical setting in question (e.g. elevated biomarkers, ischaemic signs on an electrocardiogram (ECG) and clinical presentation) *and* receiving treatment as a consequence of this (reperfusion strategies (percutaneous coronary intervention(PCI)/thrombolysis) or initiation/increased antithrombotic treatment).

Proportions of patients with clinically important GI bleeding: proportion of patients with one or more episodes of clinically important GI bleeding as defined above.

Proportion of patients with one or more infectious adverse events: proportion of patients with one or more episodes of pneumonia or CDI.

One-year mortality: landmark mortality 1 year post randomisation.

Duration of life support in the ICU: the number of days alive and free from respiratory or circulatory support and off renal replacement therapy as defined below. The outcome will be days alive without the use of mechanical ventilation, circulatory support or renal replacement therapy in the 90-day period, and will be defined as the percentage of days without mechanical ventilation, circulatory support, and renal replacement therapy (as defined in daily collected variables) in the 90 days after randomisation.

SARs: number of SARs as defined below.

The elements of all composite outcomes will be reported in the supplementary material.

A health economic analysis will be performed. The analytic details will be based on the result of the trial and specified (cost-benefit versus cost-minimisation analyses).

### Definitions of serious adverse reactions (SARs)

A SAR is defined as any adverse reaction that results in death, is life-threatening, requires hospitalisation or prolongation of existing hospitalisation, or results in persistent or significant disability or incapacity.

Patients will be monitored for onset of SARs occurring between the first dose of trial medication and until discharge from the ICU. If the patient is readmitted to the ICU and trial intervention is reintroduced, data collection for SARs will be resumed. If a patient experiences a SAR the patient will be withdrawn from the trial intervention but data collection and follow-up will be continued (see section 4.3.2).

SARs will be defined as follows:

Anaphylactic reactions defined as urticaria and at least one of the following:

- Worsened circulation (more than 20 % decrease in blood pressure or more than 20 % increase in vasopressor dose)
- Increased airway resistance (more than 20 % increase in the peak pressure on the ventilation)
- Clinical stridor or bronchospasm
- Subsequent treatment with bronchodilators

Agranulocytosis is defined as any new, acute and severe drop in granulocytes to below 0.5 × 10^9^/l requiring active monitoring or treatment.

Pancytopenia is defined as any new, severe drop in red blood cells, white blood cells and platelets requiring active monitoring or treatment.

Acute hepatic failure is defined as severe and progressing hepatic failure as judged by the treating physician or the investigator.

Stevens-Johnson syndrome and toxic epidermal necrolysis are defined as severe dermatological reactions with a skin biopsy confirming the diagnosis.

Interstitial nephritis is defined as a nephritis affecting the interstitium of the kidneys surrounding the tubules with a kidney biopsy confirming the diagnosis.

Angioedema (Quincke’s oedema) is defined as a vascular reaction involving the deep dermis, subcutaneous or submucosal tissues, resulting in a characteristic localised oedema.

## Appendix 2. Charter for the independent Data Monitoring and Safety Committee (DMSC) of the SUP-ICU trial

ClinicalTrials.gov Identifier: NCT02467621.

Research ethical committee number: H-15003141.

### Introduction

The DMSC will constitute its own plan of monitoring and meetings. However, this charter will define the minimum of obligations and primary responsibilities of the DMSC as perceived by the Steering Committee (SC), its relationship with other trial components, its membership, and the purpose and timing of its meetings. The charter will also outline the procedures for ensuring confidentiality and proper communication, the statistical monitoring guidelines to be implemented by the DMSC, and an outline of the content of the open and closed reports which will be provided to the DMSC.

### Primary responsibilities of the DMSC

The DMSC will be responsible for safeguarding the interests of trial patients, assessing the safety and efficacy of the interventions during the trial, and for monitoring the overall conduct of the clinical trial. The DMSC will provide recommendations about stopping or continuing the trial to the SC of the SUP-ICU trial. To contribute to enhancing the integrity of the trial, the DMSC may also formulate recommendations relating to the selection/recruitment/retention of patients, their management, improving adherence to protocol-specified regimens and retention of patients, and the procedures for data management and quality control.

The DMSC will be advisory to the SC. The SC will be responsible for promptly reviewing the DMSC recommendations, to decide whether to continue or terminate the trial, and to determine whether amendments to the protocol or changes in trial conduct are required.

The DMSC is planned by protocol to meet physically in order to evaluate the planned interim analyses of the SUP-ICU trial. The interim analyses will be performed by an independent statistician selected by the members of the DMSC (to be announced). The DMSC may additionally meet whenever they decide or contact each other by telephone or e-mail in order to discuss the safety of trial participants. The sponsor has the responsibility to report the overall number of serious adverse reactions (SARs) yearly to the DMSC. The DMSC can, at any time during the trial, request the distribution of events, including outcome measures and SARs according to intervention groups. Further, the DMSC can request unblinding of the interventions if suggested by the data; see section on ‘Closed sessions’. The recommendations of the DMSC regarding stopping, continuing or changing the design of the trial should be communicated without delay to the SC of the SUP-ICU trial. As soon as possible, and no longer than 48 hours later, the SC has the responsibility to inform all investigators of the trial, and all the sites including patients in the trial, about the recommendation of the DMSC and the SC decision hereof.

### Members of the DMSC

The DMSC is an independent multidisciplinary group consisting of clinicians and a biostatistician that, collectively, has experience in the management of ICU patients and in the conduct, monitoring and analysis of randomised clinical trials.

### DMSC members

Anders Åneman, MD PhD.

Tim Walsh, professor, MD, PhD.

### DMSC biostatistician

Aksel Karl Georg Jensen, Section of Biostatistics, University of Copenhagen.

### Conflicts of interest

DMSC members will fill in and sign a declaration of conflicts of interests. DMSC membership has been restricted to individuals free of conflicts of interest. The source of these conflicts may be financial, scientific, or regulatory in nature. Thus, neither trial investigators nor individuals employed by the sponsor, nor individuals who might have regulatory responsibilities for the trial products, are members of the DMSC. The DMSC members do not own stock in the companies having products being evaluated by the SUP-ICU trial.

The DMSC members will disclose to fellow members any consulting agreements or financial interests they have with the sponsor of the trial, with the Contract Research Organisation (CRO) for the trial (if any), or with other sponsors having products that are being evaluated or having products that are competitive with those being evaluated in the trial.

The DMSC will be responsible for deciding whether these consulting agreements or financial interests materially impact their objectivity.

The DMSC members will be responsible for advising fellow members of any changes in these consulting agreements and financial interests that occur during the course of the trial. Any DMSC members who develop significant conflicts of interest during the course of the trial should resign from the DMSC.

DMSC membership is to be for the duration of the clinical trial. If any members leave the DMSC during the course of the trial, the SC will appoint their replacement(s).

### Formal interim analysis meetings

Two formal interim analysis meetings will be held to review data relating to treatment efficacy, patient safety, and quality of trial conduct. The three members of the DMSC will meet when 90-day follow-up data of 1650 (approximately 50 % of sample size estimation) and 2500 (approximately 75 % of sample size estimation) patients have been obtained.

### Proper communication

To enhance the integrity and credibility of the trial, procedures will be implemented to ensure the DMSC has sole access to evolving information from the clinical trial regarding comparative results of efficacy and safety data, aggregated by treatment group. An exception will be made to permit access to an independent statistician who will be responsible for serving as a liaison between the database and the DMSC. At the same time, procedures will be implemented to ensure that proper communication is achieved between the DMSC and the trial investigators. To provide a forum for exchange of information among various parties who share responsibility for the successful conduct of the trial, a format for open sessions and closed sessions will be implemented. The intent of this format is to enable the DMSC to preserve confidentiality of the comparative efficacy results while at the same time providing opportunities for interaction between the DMSC and others who have valuable insights into trial-related issues.

### Closed sessions

Sessions involving only DMSC members who generate the closed reports (called closed sessions) will be held to allow discussion of confidential data from the clinical trial, including information about the relative efficacy and safety of interventions. In order to ensure that the DMSC will be fully informed in its primary mission of safeguarding the interest of participating patients, the DMSC will be blinded in its assessment of safety and efficacy data. However, the DMSC can request unblinding from the SC.

Closed reports will include analysis of the primary outcome measure. In addition, analyses of the secondary outcome measures and SARs will also be reported. These closed reports will be prepared by an independent biostatistician being a member of the DMSC, with assistance from the trial data manager, in a manner that allows them to remain blinded. The closed reports should provide information that is accurate, with follow-up on mortality that is complete to within 2 months of the date of the DMSC meeting.

### Open reports

For each DMSC meeting, open reports will be made available to all who attend the DMSC meeting. The reports will include data on recruitment and baseline characteristics, pooled data on eligibility violations, completeness of follow-up, and compliance. The independent statistician, being a member of the DMSC, will prepare these open reports in cooperation with the trial data manager.

The reports should be provided to DMSC members approximately 3 days prior to the date of the meeting.

### Minutes of the DMSC meetings

The DMSC will prepare minutes of their meetings. The closed minutes will describe the proceedings from all sessions of the DMSC meeting, including the listing of recommendations by the committee. Because it is possible that these minutes may contain unblinded information, it is important that they are not made available to anyone outside the DMSC.

### Recommendations to the Steering Committee

After the interim analysis meetings, the DMSC will make a recommendation to the SC to continue, hold or terminate the trial.

Interim analyses will be conducted after patient number 1650 and patient number 2500 have been followed-up for 90 days.

The DMSC will recommend pausing or stopping the trial if group difference in the primary outcome measure, SARs or SUSARs are found at the interim analyses with statistical significance levels adjusted according to the LanDeMets group sequential monitoring boundaries based on the O’Brien-Fleming alpha-spending function [48]. If an analysis of the interim data from 1650/2500 patients fulfils the LanDeMets stopping criterion the inclusion of further patients will be paused and an analysis including patients randomised during the analysis period will be performed. If this second analysis also fulfils the LanDeMets stopping criterion according to the group sequential monitoring boundaries the DMSC will recommend stopping the trial [49]. Furthermore, the DMSC can recommend pausing or stopping the trial if continued conduct of the trial clearly compromises patient safety. However, stopping for futility to show an intervention effect of 15 % RRR will not be an option as intervention effects less than 15 % RRR of all-cause mortality may also be clinically relevant.

This recommendation will be based primarily on safety and efficacy considerations and will be guided by statistical monitoring guidelines defined in this charter and the trial protocol.

The SC is jointly responsible with the DMSC for safeguarding the interests of participating patients and for the conduct of the trial. Recommendations to amend the protocol or conduct of the trial made by the DMSC will be considered and accepted or rejected by the SC. The SC will be responsible for deciding whether to continue, hold or stop the trial based on the DMSC’s recommendations.

The DMSC will be notified of all changes to the trial protocol or conduct. The DMSC concurrence will be sought on all substantive recommendations or changes to the protocol or trial conduct prior to their implementation.

### Statistical monitoring guidelines

The outcome parameters are defined in the statistical analyses plan in the protocol. For the two intervention groups, the DMSC will evaluate data on:

#### The primary outcome measure

Mortality 90 days after randomisation of each patient (‘landmark mortality’).

#### The secondary outcome measures

- Proportion of patients with one or more of the following adverse events: clinically important gastrointestinal (GI) bleeding, pneumonia, *Clostridium difficile* infection (CDI), and acute myocardial ischaemia
- Proportion of patients with clinically important GI bleeding
- One-year mortality post randomisation
- The occurrence of SARs in the ICU

The DMSC will be provided with these data from the coordinating centre as:

Number of patients randomised.

Number of patients randomised per intervention group.

Number of patients stratified pr. stratification variable per intervention group.

Number of events, according to the outcomes, in the two groups.

Based on evaluations of these outcomes, the DMSC will decide if they want further data from the coordinating centre and when to perform the next analysis of the data.

For analyses, the data will be provided in one file as described below.

The DMSC should be informed yearly about SARs occurring in the two groups of the trial.

The DMSC may also be asked to ensure that procedures are properly implemented to adjust trial sample size or duration of follow-up to restore power if protocol-specified event rates are inaccurate. If so, the algorithm for doing this should be clearly specified.

### Conditions for transfer of data from the coordinating centre to the DMSC

The DMSC will be provided with a file containing the data defined as follows:

Row 1 contains the names of the variables (defined below).

Row 2 to *N* (where *N −* 1 is the number of patients having entered the trial) each contains the data of one patient.

Column 1 to *p* (where *p* is the number of variables to be defined below) each contains in row 1 the name of a variable and in the next *N* rows the values of this variable.

The values of the following variables should be included in the database:

1. screening_id: a number that uniquely identifies the patient
2. rand_code: the randomisation code (group 0 or 1). The DMSC is not to be informed on what intervention the groups received
3. clin_imp_bleed: clinically important GI bleeding (1 = the patient had one or more episodes, 0 = the patient did not)
4. pneumonia: onset of pneumonia in the ICU after randomisation (1 = one or more episodes, 0 = no episodes)
5. clostridium: *Clostridium difficile* infection (1 = one or more episodes, 0 = no episodes)
6. ami: acute myocardial ischemia in the ICU (1 = one or more episodes, 0 = no episodes)
7. SAR: SAR indicator (1 = one or more SARs, 0 = no SARs).

## Appendix 3. Power estimations

All power estimations have been calculated on data from the international 7-day inception cohort study [9].

Since we do not know whether treatment with acid suppressants reduce or increase mortality, a number of scenarios have been considered (±20 relative risk reduction):

1. 25.0 % mortality 90 days after inclusion among patients with:

- At least one risk factor*.
- No acid suppressants at ICU admission.
- Treatment with acid suppressants during ICU admission.
- No clinically important bleeding** during ICU admission.

### Power estimation

**Table:**

ARR	Power	Patients per group
−5 %	80 %	1091
	90 %	1461
+5 %	80 %	1248
	90 %	1671

*ARR* absolute risk reduction

We do not know whether a PPI benefits or harms the patients, and need to include both scenarios. With 1671 patients in each group we will be able to show an absolute increase in risk of 5 % with 90 % power at the primary outcome, but also an absolute risk reduction of 5 % with 90 % power.

The sample size has been calculated on patients fulfilling inclusion and exclusion criteria in the SUP-ICU trial and because few patients were not treated with acid suppressants during ICU admission, the estimation is based on the group receiving acid suppressants (intervention group).

2. 25.9 % mortality 90 days after inclusion among patients with:

- At least one risk factor*.
- No acid suppressants at ICU admission.
- Treatment with acid suppressants during ICU admission.
- Bleeding (overt or clinically important**) or no bleeding during ICU admission.

### Power estimation

**Table:**

ARR	Power	Patients per group
−5.2 %	80 %	1034
	90 %	1384
+5.2 %	80 %	1180
	90 %	1579

*ARR* absolute risk reduction

3. 29.2 % mortality 90 days after inclusion among patients with:

- At least one risk factor*.
- Acid suppressants and no acid suppressants at ICU admission.
- Treatment with acid suppressants during ICU admission.
- No bleeding (overt or clinically important**) during ICU admission.

### Power estimation

**Table:**

ARR	Power	Patients per group
−5.8 %	80 %	901
	90 %	1206
+5.8 %	80 %	1014
	90 %	1357

*ARR* absolute risk reduction

4. 30.5 % mortality 90 days after inclusion among patients with:

- At least one risk factor*.
- Acid suppressants or no acid suppressants at ICU admission.
- Treatment with acid suppressants during ICU admission.
- Bleeding (overt or clinically important**) or no bleeding during ICU admission.

### Power estimation

**Table:**

ARR	Power	Patients per group
−6.1 %	80 %	837
	90 %	1120
+6.1 %	80 %	937
	90 %	1254

*ARR* absolute risk reduction

*Risk factors are: shock, renal replacement therapy, coagulopathy, and coagulopathy and liver disease as comorbidities.

**Overt bleeding is defined as any episode of haematemesis, coffee ground emesis, melaena, haematochezia or bloody nasogastric aspirate.

Clinically important bleeding is defined as overt bleeding and at least one of the following four features within 24 hours of GI bleeding (in the absence of other causes) [1, 5] in the ICU:

- Spontaneous drop of systolic blood pressure, mean arterial pressure or diastolic blood pressure of 20 mmHg or more
- Start of vasopressor or a 20 % increase in vasopressor dose
- Decrease in haemoglobin of at least 2 g/dl (1.24 mmol/l)
- Transfusion of two units of packed red blood cells or more.

## Abbreviations

CDI: *Clostridium difficile* infection
CRF: case report form
CRIC: Centre for Research in Intensive Care
CT: computed tomography
CTSM: Clinical Trial Supply Management
CTU: Copenhagen Trial Unit
DASAIM: Danish Society of Anaesthesiology and Intensive Care Medicine
DMSC: Data Monitoring and Safety Committee
eCRF: electronic case report form
GCP: Good Clinical Practice
GI: gastrointestinal
H2RA: histamine-2-receptor antagonist
HIV: human immunodeficiency virus
hCG: human chorionic gonadotropin
ICU: intensive care unit
INR: international normalised ratio
PPI: proton pump inhibitor
PT: prothrombin time
RCT: randomised clinical trial
RRI: relative risk increase
RRR: relative risk reduction
RRT: renal replacement therapy
SAE: serious adverse event
SAR: serious adverse reaction
SCCTG: Scandinavian Critical Care Trial Group
SSAI: Scandinavian Society of Anaesthesia and Intensive Care Medicine
SUP: stress ulcer prophylaxis
SUSAR: severe unexpected serious adverse reaction
TSA: trial sequential analysis
